# A Wavelet Packets Approach to Electrocardiograph Baseline Drift Cancellation

**DOI:** 10.1155/IJBI/2006/97157

**Published:** 2006-11-20

**Authors:** Mohammad Ali Tinati, Behzad Mozaffary

**Affiliations:** Communication Department, Faculty of Electrical and Computer Engineering, University of Tabriz, Tabriz 51666-16471, Iran

## Abstract

Baseline wander elimination is considered a classical problem. In electrocardiography (ECG) signals, baseline drift can influence the accurate diagnosis of heart disease such as ischemia and arrhythmia. We present a wavelet-transform- (WT-) based search algorithm using the energy of the signal in different scales to isolate baseline wander from the ECG signal. The algorithm computes wavelet packet coefficients and then in each scale the energy of the signal is calculated. Comparison is made and the branch of the wavelet binary tree corresponding to higher energy wavelet spaces is chosen. This algorithm is tested using the data record from MIT/BIH database and excellent results are obtained.


Hindawi Publishing Corporation
International Journal of Biomedical Imaging
Volume 2006, Article ID 97157, Pages 1–9
DOI 10.1155/IJBI/2006/97157

### A Wavelet Packets Approach to Electrocardiograph Baseline Drift Cancellation

**Mohammad Ali Tinati and Behzad Mozaffary**

*Communication Department, Faculty of Electrical and Computer Engineering, University of Tabriz, Tabriz 51666-16471, Iran*

Received 29 October 2005; Revised 31 August 2006; Accepted 11 September 2006

Recommended for Publication by Scott Pohlman

Baseline wander elimination is considered a classical problem. In electrocardiography (ECG) signals, baseline drift can influence the accurate diagnosis of heart disease such as ischemia and arrhythmia. We present a wavelet-transform- (WT-) based search algorithm using the energy of the signal in different scales to isolate baseline wander from the ECG signal. The algorithm computes wavelet packet coefficients and then in each scale the energy of the signal is calculated. Comparison is made and the branch of the wavelet binary tree corresponding to higher energy wavelet spaces is chosen. This algorithm is tested using the data record from MIT/BIH database and excellent results are obtained.

Copyright © 2006 M. A. Tinati and B. Mozaffary. This is an open access article distributed under the Creative Commons Attribution License, which permits unrestricted use, distribution, and reproduction in any medium, provided the original work is properly cited.

### 1. INTRODUCTION

In recent years computer aided ECG signal analysis has gained momentum and a tremendous amount of work has been carried out. One area of interest has been removing artifacts from data records [1, 2]. Artifacts are the noise induced to ECG signals that result from movements of electrodes. This in turn causes deformation and change in the electrical characteristics of the skin under and around the electrodes. These electrical changes appear in the ECG as motion artifacts and baseline drifts. Baseline wanders are considered as an artifact which produces inaccurate data when measuring the ECG parameters. The ST-segment measures are especially strongly affected by this wandering. In most of the ECG recordings the respiration, electrode impedance changes due to perspiration and increased body movements are the main causes of the baseline wandering [3]. Therefore, elimination of the baseline drifts can very much change the clinical information of the ECG signal.

The frequency components of the baseline wander are usually below 0.5 Hz which is higher under stress test condition. Baseline removal has been addressed in many different ways in literature. Ensemble averaging is a classical statistical method for baseline cancellation. This is not an accurate way since characteristics of ECG signal change from beat to beat. Therefore, the consequences of mean-based algorithms are ST displacements.

In [4], baseline estimation method using cubic spline which is a portion of the Maclaurin series (higher than the 4th derivatives are neglected) is proposed. This is a third order approximation where the baseline is estimated by polynomial approximations and then subtracted from the original raw ECG signal. This is a nonlinear method, and its performance is based on estimation of reference points in the PR intervals. The main disadvantage of this method is estimating reference points that may not belong to baseline.

In [5], a linear time-varying filtering approach is undertaken to suppress the baseline drift in the ECG signal. Beat average is subtracted from the signal and then decimated. Low-pass filtering is applied to estimate the baseline wander and is interpolated. Then it is subtracted from the original signal. This is a nonlinear approach so it is complex and highly dependent to beat rate calculations and becomes less accurate in low heart rates.

Another linear filtering method has been suggested in [6]. In this approach a digital narrowband linear-phase FIR filter with cut-off frequency of 0.8 Hz is applied. Although it can be implemented in real time, this approach has many serious problems. Another filtering technique using digital and hybrid linear-phase with cut-off frequency of 0.64 Hz is used in [7]. There are numerous problems associated with filtering. First, when the FIR structure is used, the number of coefficients is too high and therefore results in a long impulse

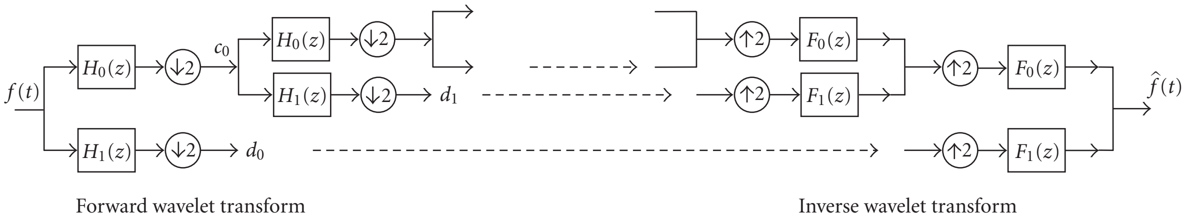

Figure 1: Filter bank representation of wavelet decomposition.

response results; second, there is an overlap on the spectral range of ECG and baseline and any cut-off frequency above 0.05 Hz does not comply with the AHA recommendation for ECG recordings [8], and removing them from ECG will introduce distortion on the ST segment as well as the QRS complex.

Adaptive filtering has been shown to be useful in many areas of biomedical signal processing, where baseline wander elimination is not an exception. Application of adaptive filters in baseline drift removal can be considered as notch filtering with lower cut-off frequency being at zero. In [9] a single tap adaptive filter with a constant reference signal (DC signal) is proposed. Although it effectively removes baseline, it produces some distortion in the ST-segment due to attenuation of low-frequency components of the ECG signal. A cascade of adaptive filters is used in [10]. The first stage is the replica of the filter scheme used in [9] with a small improvement in the bandwidth. In the second stage, the reference input is changed to an impulse sequence obtained from the QRS complex. Another drawback in these filters is slow tracking properties; therefore, a smoothed ECG signal will result.

Transform techniques have been also used to remove baseline drifts. In [11], it is suggested to use short time Fourier transform (STFT) to detect the presence of baseline drifts in the ECG signal and then use a time-varying filter similar to ones used in [5] to remove it. This method has two problems. First, STFT cannot have good time and frequency resolution at the same time. Second, it will have the same problems that are associated with filtering that is already mentioned above. Discrete wavelet transform (DWT) has also been used in baseline wander removal. In [12], baseline wander is estimated from the DWT course level coefficients at level j, and then it was subtracted from the original ECG signal. In this method Symlets wavelet of the order 10 was used. In [13], wavelet coefficients are calculated up to the sixth resolution level. Then the energy levels of the first and last levels are compared. Up to two stages of filtering are carried out based on the energy levels of the wavelet coefficients.

In this paper, a wavelet-transform-based method to remove baseline wander in ECG signals is described. This method is robust to noise and is based on estimation of the energy of the baseline and it is removed through inverse discrete wavelet transform. In Section 2 a brief introduction to wavelets, its properties, and multiresolution analysis is given. In Section 3, ECG signal characteristic points related to various activities of heart are explained. In Section 4 the proposed method is described and its effectiveness is examined. In Section 5 the MIT-BIH ECG database records are used to evaluate the performance of the proposed algorithm. In Sections 6 and 7 discussions and conclusion are made.

### 2. WAVELETS

Wavelets are transform methods that have received great deal of attention in the field of signal processing over the past several years. A wavelet system is a set of building blocks from which one can construct or represent a signal or a function. It is a two-dimensional expansion set. The wavelet transform is a time-scale representation method that decomposes signal $f(t)$ into basis functions of time and scale which are dilated and translated versions of a basis function $\psi(t)$ which is called mother wavelet [14]. Translation is accomplished by considering all possible integer translations of $\psi(t)$ and dilation is obtained by multiplying $t$ by a scaling factor which is usually factors of 2. The following equation shows how wavelets are generated from the mother wavelet:

$$\psi_{j,k}(t) = 2^{j/2}\psi(2^{j/2}t - k), \qquad (1)$$

where $j$ indicates the resolution level and $k$ is the translation in time. This is called dyadic scaling, since the scaling factor is taken to be 2.

Wavelet decomposition is a linear expansion and it is expressed as

$$f(t) = \sum_{k=-\infty}^{+\infty} c_k \varphi(t-k) + \sum_{k=-\infty}^{+\infty} \sum_{j=0}^{+\infty} d_{j,k}\psi(2^j t - k), \qquad (2)$$

where $\varphi(t)$ is called the scaling function or father wavelet and $c_k$ and $d_{j,k}$ are the coarse and detail level expansion coefficients, respectively.

In the field of signal processing, most of the results of wavelet theory are developed using filter banks. In applications one never has to deal directly with the scaling functions or wavelets, only the coefficients of the filters in the filter banks are needed. In the forward wavelet transform system, the signal is convolved with a pair of maximally decimated quadrature mirror filters (QMF) [14, 15]. Let $H_0(z)$ and $H_1(z)$ be low-pass and high-pass filters, respectively, that split the signal's bandwidth to half. These filters along with decimators manipulate the forward wavelet transform. The wavelet decomposition system is shown in Figure 1. In this

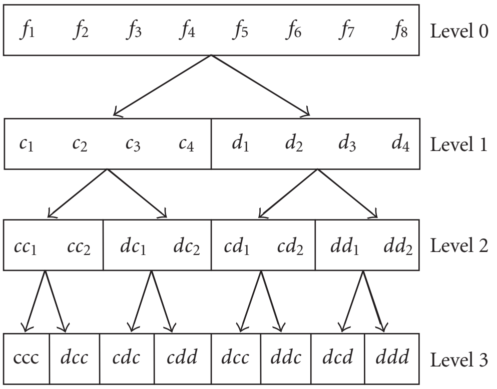

Figure 2: Wavelet packet decomposition tree.

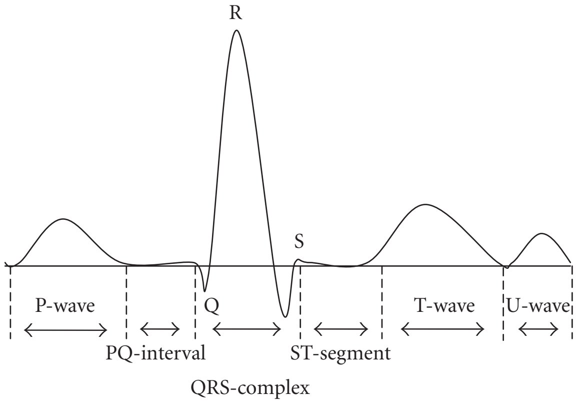

Figure 3: A typical ECG signal.

figure, filters $F_0(z)$ and $F_1(z)$ and the interpolator perform the reverse process in order to reconstruct the original signal $f(t)$. These filters are related to wavelet and scaling functions as expressed below [16]:

$$\psi(t) = \sum_k \sqrt{2}h_1(k)\varphi(2t-k),$$
$$\varphi(t) = \sum_k \sqrt{2}h_0(k)\varphi(2t-k). \quad (3)$$

In biorthogonal filter banks, the impulse responses of synthesis filters are related to analysis filters as

$$g_0(n) = (-1)^n h_0(1-n),$$
$$g_1(n) = (-1)^n h_1(1-n). \quad (4)$$

Theoretically, in (2) the expansion coefficients $c_k$ and $d_{j,k}$ are calculated from the inner product of $f(t)$ with $\varphi(t)$ and $\psi(t)$, respectively. The power of wavelet transform is based on the fact that these coefficients are computed in a recursive manner. Once $c_k$ is known in a starting scale $J$, all the coefficients for $j = J, J-1, \dots,$ are found by a simple linear transform.

A wide variety of functions could be chosen as the mother wavelet as long as (5) is satisfied:

$$\int_{-\infty}^{+\infty} \psi(t)dt = 0. \quad (5)$$

Wavelets are useful in applications such as signal denoising, wave detection, data compression, feature extraction, and so forth. There are many techniques based on wavelet theory, such as wavelet packets, wavelet approximation and decomposition, discrete and continuous wavelet transform, and so forth.

In Figure 1 only one-sided expansion of the signal using coarse information was shown. This is not completely satisfactory, and more flexibility is obtained in full wavelet packet decomposition. A full binary tree for tree scale wavelet packet transform is shown in Figure 2. The projection of the function on the scaling functions and the wavelets are recursively decomposed to obtain the binary tree.

As Figure 2 shows, at each level both high-pass and low-pass filters are applied followed by decimation where both coarse and detail level coefficients are obtained. In this figure "$c$" and "$d$" stand for course and details of the signal, respectively. This configuration gives possibility of many different paths for decomposition.

The energy of a signal is given in terms of the wavelet coefficients by Parsaval's relation as

$$\int |f(t)|^2 dt = \sum_{l=-\infty}^{\infty} |c_l|^2 + \sum_{j=0}^{\infty} \sum_{k=-\infty}^{\infty} |d_{jk}|^2. \quad (6)$$

We will use this equation for computing the energy of the signal in different scales, where it is explained more in later sections.

### 3. ECG SIGNAL CHARACTERISTICS

In a typical ECG signal, the dominant parts are considered as P-wave, P-R interval, QRS complex, ST-segment, T-wave, TU-segment, U-wave, and UP-segment. In Figure 3 a typical ECG signal is shown. The P-wave represents both of the artria activation or depolarization. The first half of the P-wave is the activation of the right artrium, while the second half is the activation of the artria septum and left artrium. The QRS complex represents the ventricular depolarization. The Q-wave is the first negative deflection right after the P-wave. The R-wave is the first positive deflection after the P-wave. The S-wave is the first negative deflection after the R-wave. The T-wave is the ventricular recovery or repolarization [17].

ST-segment has clinical significance in diagnosis of myocardial ischemia and infarction. Slow fluctuation of baseline wander can influence the ST-segment and therefore incorrect diagnosis may happen. In addition, accurate baseline detection is needed for the localization of ventricular arrhythmias with body surface potential mappings as the major frequency components of ST-segment and baseline drifts are close.

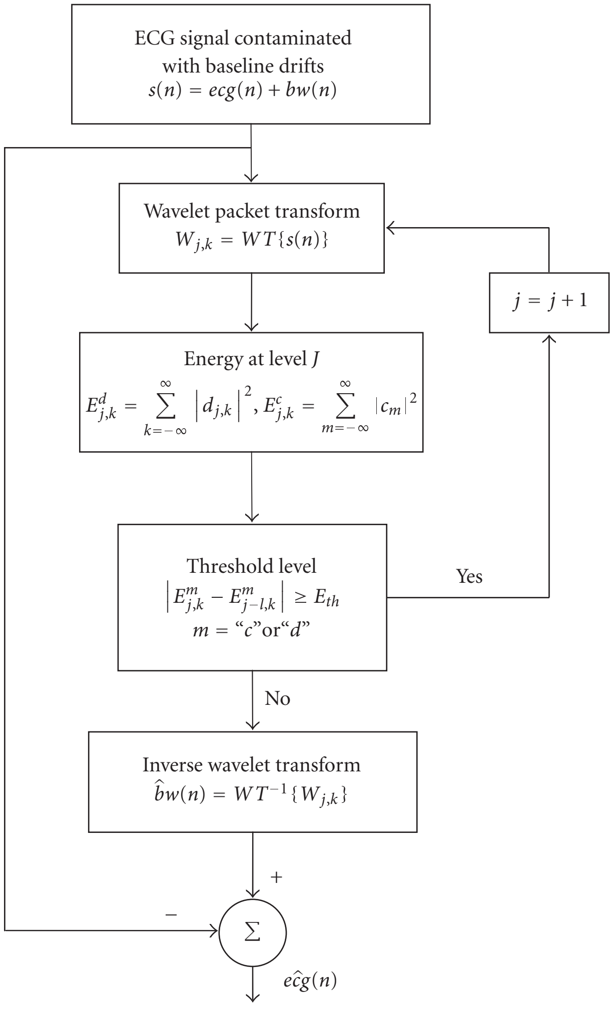

Figure 4: Block diagram of ECG baseline remover algorithm.

### 4. METHOD

In this section we discuss a new algorithm that we propose for removal of the baseline wander in ECG signals. The morphological features of the ECG signal often change by various sources, such as 50 Hz power line, motion artifacts baseline drifts, and so forth. It is of great importance for diagnosis purposes to remove the baseline wandering, especially in the case of precise measurement of the ST-segment. This segment is a slow varying part of the ECG signal where parts of its spectral components overlap with spectral components of baseline wandering. Therefore, by simple digital filtering it is not possible to remove baseline drifts.

Our proposed algorithm is based on the assumption that the baseline drift and ECG signal constitute a mixture of two independent signals, mixed in a linear fashion. Figure 4 shows the block diagram of the procedure. First, the wavelet transform of this signal is computed. The dyadic wavelet packet decomposition of the ECG signal is carried out using Daub-4 mother wavelet. It is well known that high-frequency components are mostly focused on low-level scales.

Therefore, it is expected to observe the baseline drift in larger scales.

In each scale using the wavelet coefficients, the energies of the signal for both the coarse and detail levels are calculated. These energies represent the energy of the decomposed signal in assumed scales as

$$E^c_{j,k} = \sum_{m=-\infty}^{\infty} \left| c_m \right|^2,$$
$$E^d_{j,k} = \sum_{k=-\infty}^{\infty} \left| d_{j,k} \right|^2. \tag{7}$$

In the above equations, $E^c_{j,k}$ is the energy of the signal in the coarse level of scale $j$ (low-pass filtering branch), and $E^d_{j,k}$ is the energy in the detail level of the signal at scale $j$ (high-pass filtering branch). In Figure 5, the power spectrum density of the ECG signal for the MIT-BIH record "103" is shown. As the figure shows, concentrations of the energy at high frequencies decrease as the scale is increased. In this figure, the wavelet coefficients $d_0$ to $d_8$ which are the details of the signal at corresponding levels, and the coefficient $c_8$ is the coarse or the approximation at the last scale, are plotted.

The next step in the algorithm is to compare these energy levels and then choose the branch of the binary tree that has the higher energy. In this step, best basis functions for decomposition are chosen. The path for higher energy branches will be followed until a point is reached where energy differences exceed a preset threshold level, $E$th. In this point the binary tree search is completed, and the baseline wander signal is retrieved by taking the inverse wavelet transform of the wavelet packet coefficients of the last scale. In order to suppress the baseline drift, the estimated baseline wander is subtracted from the original data record and a baseline wander free ECG signal is identified.

Selecting the threshold level is accomplished in the following manner. The normalized energy and normalized bandwidth of the signal are calculated. In every scale, the product of bandwidth and energy is obtained. It was experimentally determined that whenever this value is 0.1% of the total energy-bandwidth product of the signal, then the last scale has been reached.

### 5. SIMULATION RESULTS

In this section, examples are presented in order to illustrate the effectiveness of the proposed algorithm. Removing the baseline wander using the above concept was tested on ECG data records. The test database was extracted from the MIT-BIH database. Records "b108," "b210," and "b115" which are severely distorted by baseline wandering were used. Each data is 50 seconds in duration with 11 b/sample resolution and 360 Hz sampling rate. Results are shown in Figures 6 to 8. This algorithm was tested on 15 other data records from MIT-BIH, and was successfully able to remove the baseline drifts efficiently from all of the signals. In addition to tests carried out on these real data records, a low-frequency sine-wave (0.01 Hz) was also added to a baseline free ECG

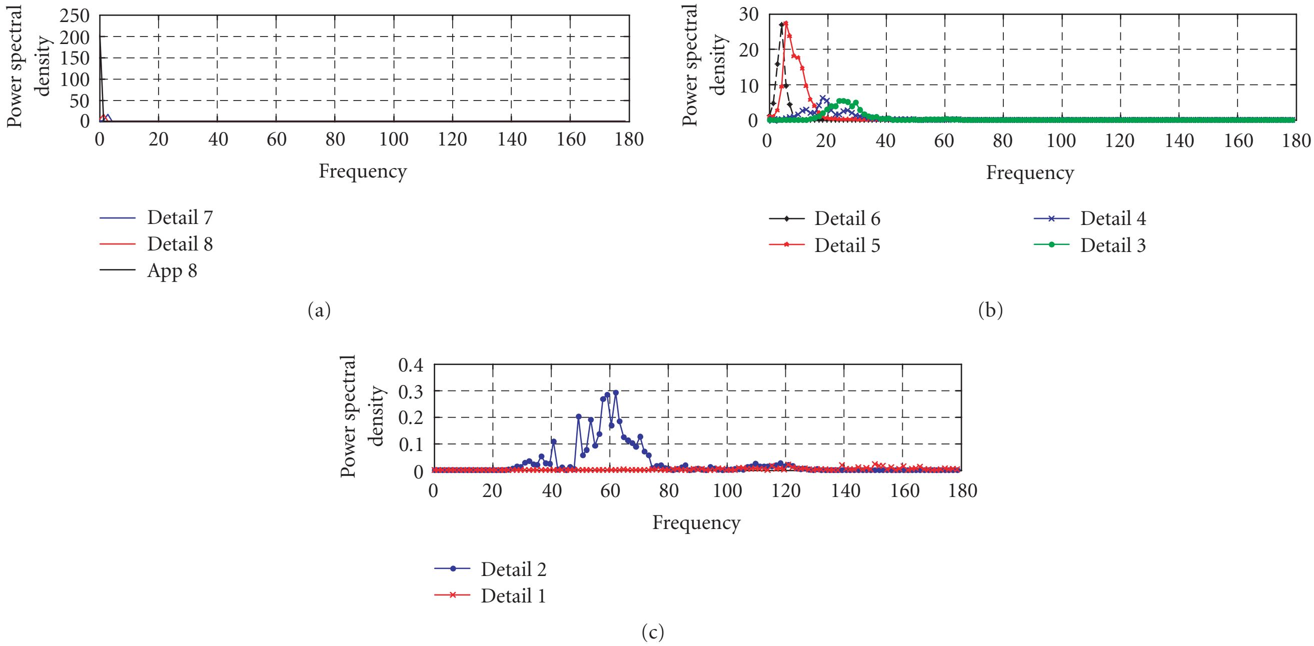

Figure 5: Power spectrum density of ECG at $2^{-1}$ to $2^{-8}$ scales.

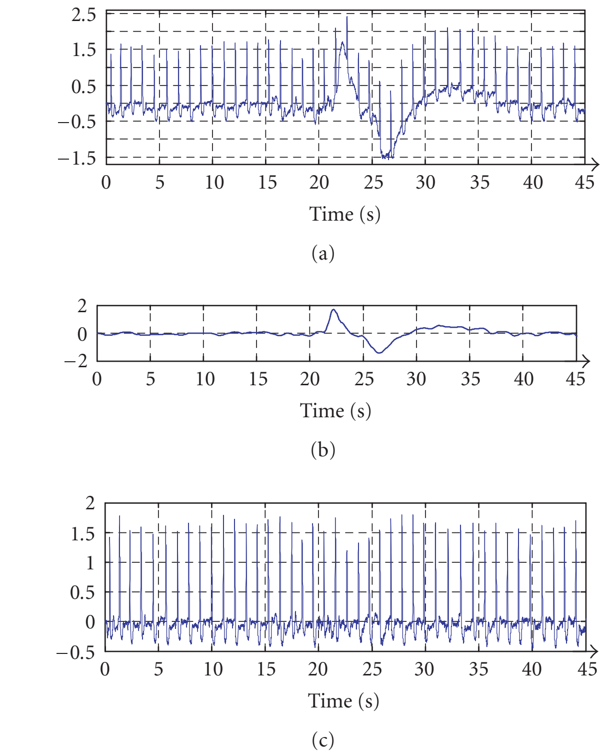

Figure 6: The ECG record "b108-2," (a) original ECG signal, (b) estimated baseline drift, (c) ECG after removing the baseline.

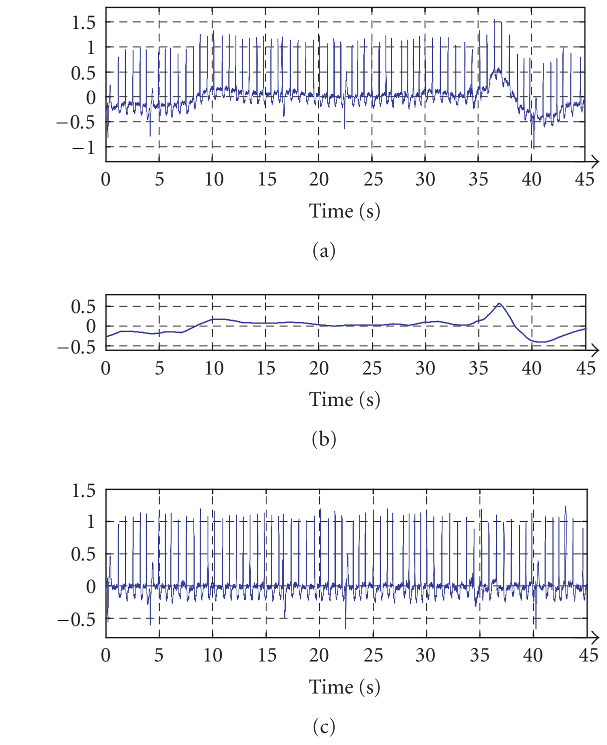

Figure 7: The ECG record "b210-1," (a) original ECG signal, (b) estimated baseline drift, (c) ECG after removing the baseline.

signal with a ratio of ECG signal power to baseline power of −5 dB, and then algorithm was applied. Results are shown in Figure 9, which indicates that exactly the added signal was removed. This is confirmed by calculating the PRD of the added sine-wave as

$$\mathrm{PRD} = \frac{\sqrt{f(t)^2 - \hat{f}(t)^2}}{f(t)}, \tag{8}$$

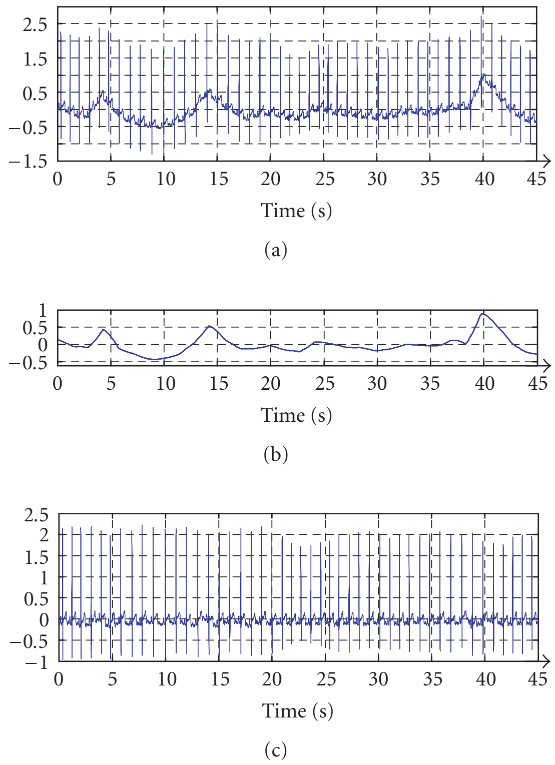

Figure 8: The ECG record "b115-1," (a) original ECG signal, (b) estimated baseline drift, (c) ECG after removing the baseline.

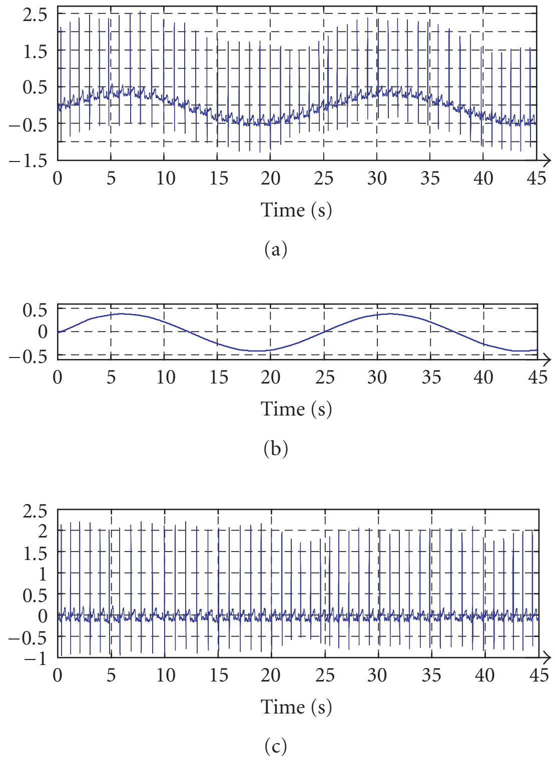

Figure 9: Baseline removal from ECG plus sine-wave. (a) ECG signal with added sine-wave, (b) estimated baseline drift, (c) ECG after removing the baseline.

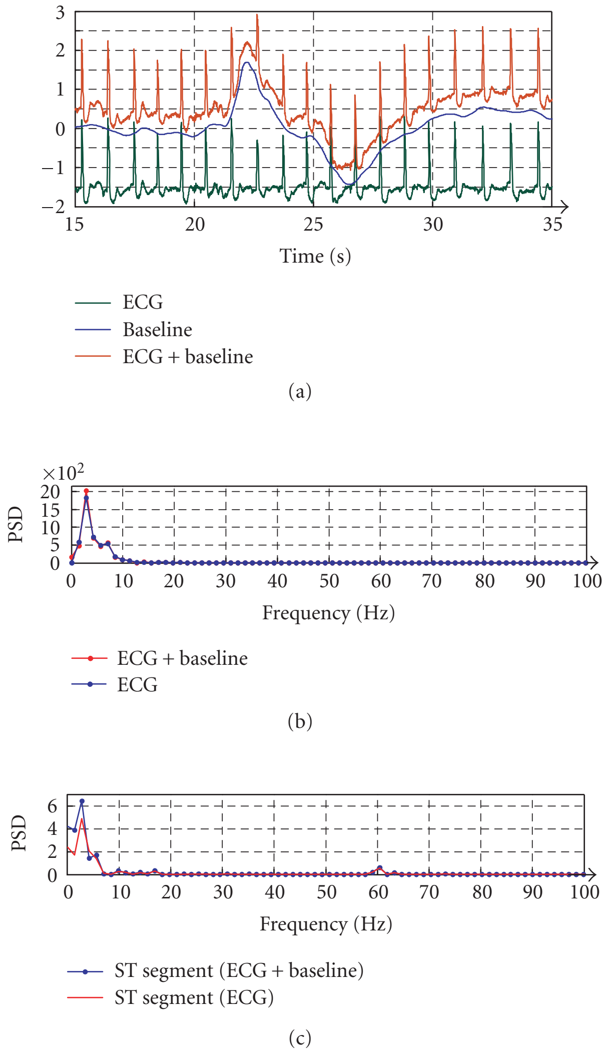

Figure 10: (a) 20 seconds of record "b108-2" highlighted, (b) power spectrum density of record "b108-2," and (c) power spectrum density of ST-segments of record "b108-2."

where $f(t)$ is the added sine-wave and $\hat{f}(t)$ is the estimated one. The value of the PRD is 0.0199, which show very good estimation of $f(t)$.

As the pictorial evaluations of the above-mentioned figures show, baseline drifts were successfully removed. In order to see if the clinical features of ECG signals are distorted or not, the spectrums of the ST-segments are evaluated. First, ST-segments were separated from all of the test signals. Power spectrum density (PSD) of ST-segments before and after applying the algorithm are calculated. Results are shown in Figures 10(c) to 12(c). These figures indicate that low-frequency power levels were reduced while the energies of ST-segments are preserved. In parts (b) of these figures, the PSDs of whole data records before and after removing the baseline are shown. As figures indicate, the high-frequency components of signals were not affected at all. In order to clarify the effectiveness of the algorithm, in part (a)

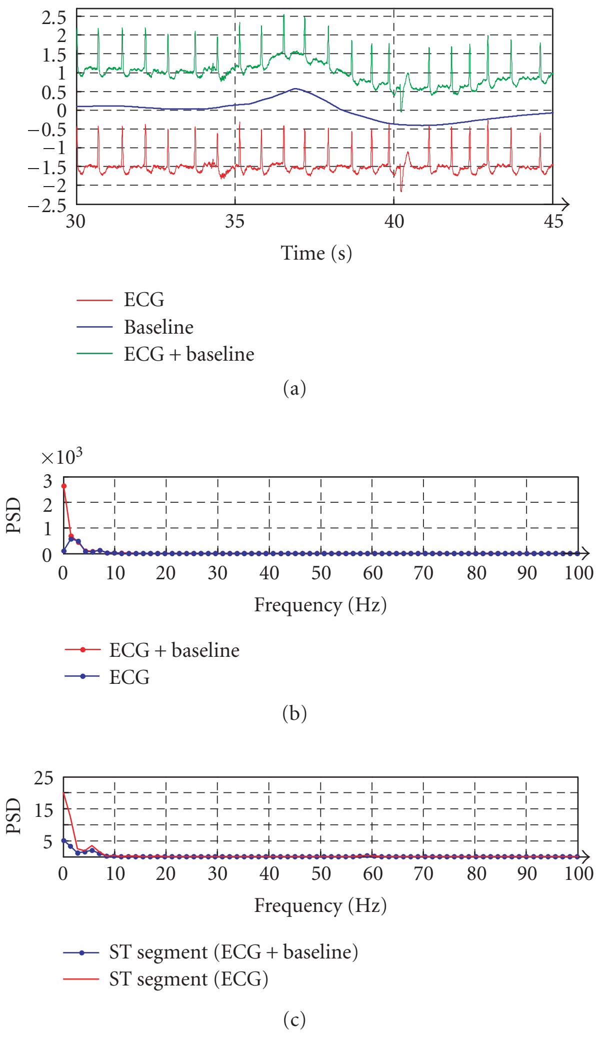

Figure 11: (a) 15 seconds of record “b210-1” highlighted, (b) power spectrum density of record “b210-1,” (c) power spectrum density of ST-segments of record “b210-1.”

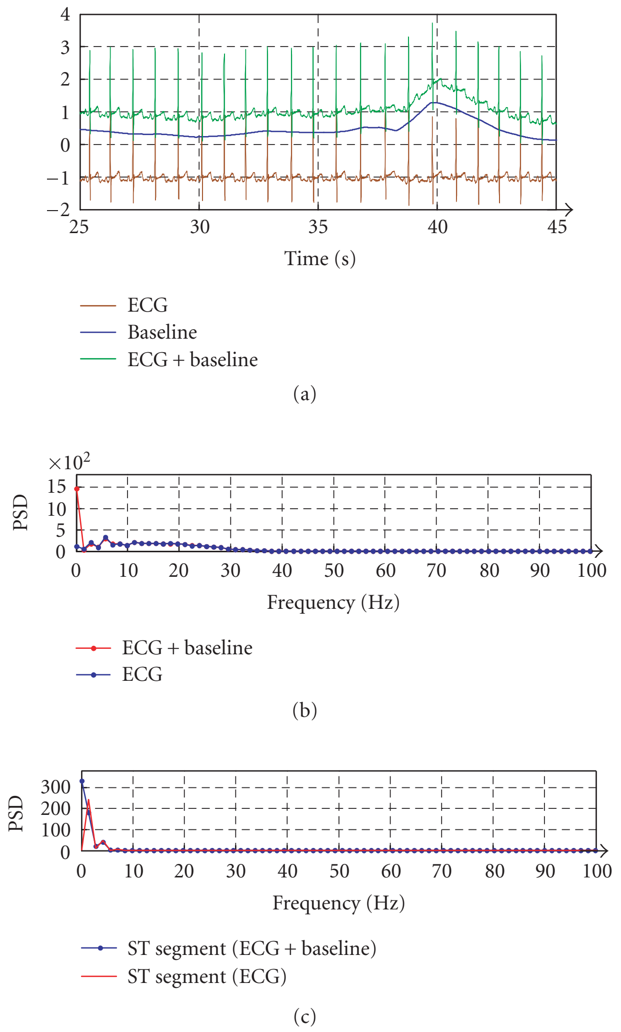

Figure 12: (a) 20 seconds of record “b115-1” highlighted, (b) power spectrum density of record “b115-1,” (c) power spectrum density of ST-segments of record “b115-1.”

of these figures, those sections of ECG signals of Figures 6 to 8 that are corrupted mostly with baseline drift are highlighted. Note that in these figures, both the original ECG signal and ECG with the removed baseline drift as well as the estimated baseline are shown. This analysis is carried out for the signal of Figure 9 and results are shown in Figure 13.

We have also compared R-R intervals before and after applying the algorithm. Any change in R-R interval will cause distortion in the ST-segment and as a result clinical information of the ECG signal will change. We first identified QRS peaks of ECG records using a peak detector scheme, and then time differences between the peaks are evaluated. Results are depicted in Figure 14, which shows R-R intervals exactly before and after coinciding. Note that graphs are plotted apart from each other for comparison purposes.

In the implementation of the wavelet packet transform, in the construction of the filter bank, Daubechies $db_4$ mother wavelet was used.

### 6. DISCUSSIONS

There are number of advantages in using wavelet transfer method in the analysis of ECG signals. First, there is no need for a priori information about the signal being processed. The second advantage is its easy implementation. The ability of the proposed wavelet-based algorithm was demonstrated by test signals. This algorithm could be used in multiple ECG recordings, especially in stress tests. An additional advantage of using wavelets would be utilization of WT in high-frequency noise cancellations. Daubechies wavelets are suitable for ECG signal analysis because they bear some morphological resemblance to them.

The proposed method removes the components that are not correlated to ECG signal and has such characteristics that somehow are added to it. Since the spectrum of the baseline is below the spectrum of the ECG signal, therefore its energy concentration in corresponding time-scale plane does

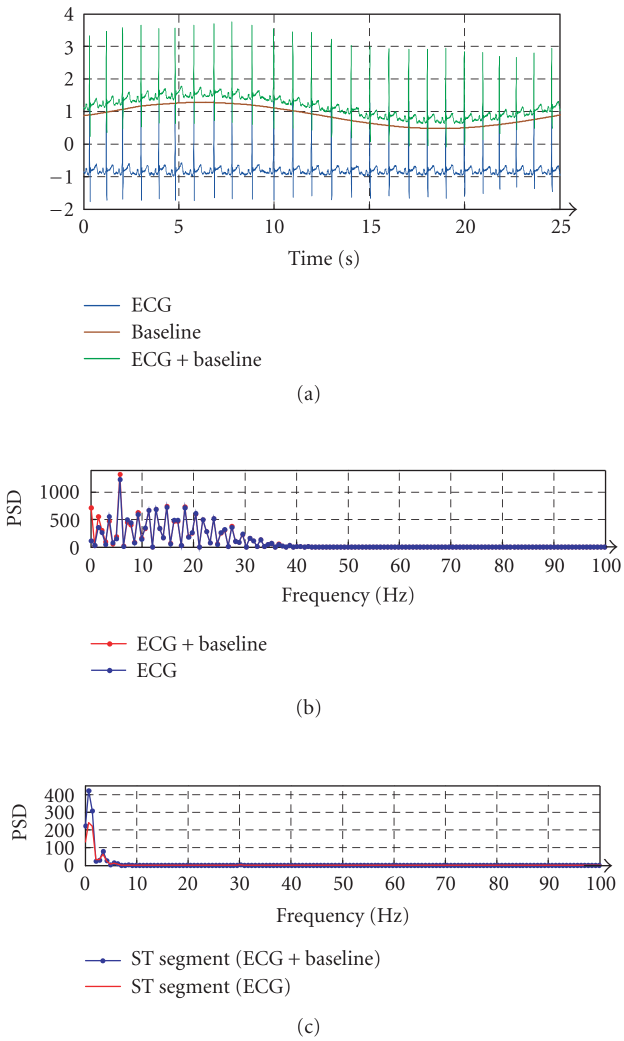

FIGURE 13: (a) 25 seconds of sine-wave plus clean ECG highlighted, (b) power spectrum density of the signal, (c) power spectrum density of ST-segments of the signal.

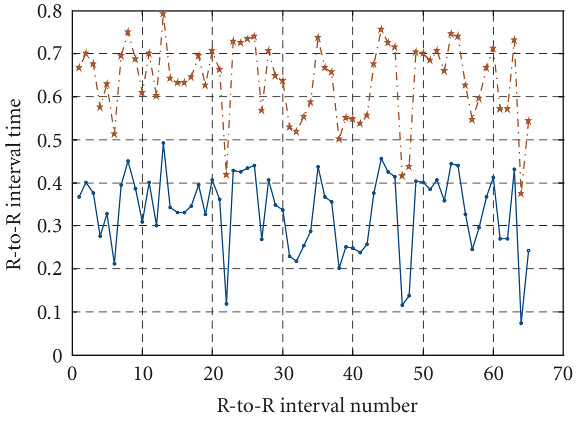

FIGURE 14: A typical R-R interval before and after removing the baseline wander; top graph represents before removal and the other one is after removal.

not change much as the scale is changed in the binary tree, but the energy of the ECG signal decreases as the scale is increased. Therefore, in the binary tree search, we reach the point that the energy of the ECG signal almost vanishes (no details in that scale) but we still have considerable energy for baseline wander.

In removing the baseline wander from ECG signal, the clinical information of the ECG record must be preserved. The most important part of the ECG signal that could be affected by the baseline is the ST-segment, whose spectrums overlap. R-R interval change could be another source of distortion. Both of these have been examined using PSD calculations.

The main cause of error in our analysis could be in determining the ST-segment's length for PSD calculations. This is not fully solved because of the complicated structure of the signal.

Although the proposed method is applied only for ECG baseline suppression, it could be used in any other biological signal processing cases for unwanted low-frequency cancellation. We used this method in off-line preprocessing of the ECG signal, it could be used in real-time applications as well.

In the current study we have used a fixed threshold level to identify the energy level of the baseline wander in wavelet domain (*E*th in Figure 4). Further study can be carried out for an adaptive threshold determination. Another point of further exploration could be the use of other mother wavelets such as biorthagonal wavelets.

### 7. CONCLUSION

In this paper we presented an algorithm based on WT for removing baseline drifts in ECG signals. It has been shown that the presented algorithm can eliminate baseline drifts from ECG signals without introducing any deformation to the signal and losing any clinical information.

### REFERENCES

[1] D. A. Tong, K. A. Bartels, and K. S. Honeyager, "Adaptive reduction of motion artifact in the electrocardiogram," in *Proceedings of the Second Joint Engineering in Medicine and Biology, 24th Annual Conference and the Annual Fall Meeting of the Biomedical Engineering Society (EMBS/BMES '02)*, vol. 2, pp. 1403–1404, Houston, Tex, USA, October 2002.

[2] P. S. Hamilton, M. G. Curley, R. M. Aimi, and C. Sae-Hau, "Comparison of methods for adaptive removal of motion artifact," in *Proceedings of Computers in Cardiology*, pp. 383–386, Cambridge, Mass, USA, September 2000.

[3] G. M. Friesen, T. C. Jannett, M. A. Jadallah, S. L. Yates, S. R. Quint, and H. T. Nagle, "A comparison of the noise sensitivity of nine QRS detection algorithms," *IEEE Transactions on Biomedical Engineering*, vol. 37, no. 1, pp. 85–98, 1990.

[4] C. R. Meyer and H. N. Keiser, "Electrocardiogram baseline noise estimation and removal using cubic splines and state-space computation techniques," *Computers and Biomedical Research*, vol. 10, no. 5, pp. 459–470, 1977.

[5] L. Sornmo, "Time-varying filtering for removal of baseline wander in exercise ECGs," in *Proceedings of Computers in Cardiology*, pp. 145–148, Venice, Italy, September 1991.

[6] J. A. Van Alste and T. S. Schilder, "Removal of base-line wander and power-line interference from the ECG by an efficient FIR filter with a reduced number of taps," *IEEE Transactions on Biomedical Engineering*, vol. 32, no. 12, pp. 1052–1060, 1985.

[7] I. I. Christov, I. A. Dotsinsky, and I. K. Daskalov, "High-pass filtering of ECG signals using QRS elimination," *Medical and Biological Engineering and Computing*, vol. 30, no. 2, pp. 253–256, 1992.

[8] H. V. Pipberger, R. C. Arzbaecher, A. S. Berson, et al., "Recommendations for standardization of leads and of specifications for instruments in electrocardiography and vectorcardiography," *Circulation*, vol. 52, pp. 11–31, 1975.

[9] N. V. Thakor and Y.-S. Zhu, "Applications of adaptive filtering to ECG analysis: noise cancellation and arrhythmia detection," *IEEE Transactions on Biomedical Engineering*, vol. 38, no. 8, pp. 785–794, 1991.

[10] P. Laguna, R. Jane, and P. Caminal, "Adaptive filtering of ECG baseline wander," in *Proceedings of the Annual International Conference of the Engineering in Medicine and Biology Society (EMBS '92)*, vol. 2, pp. 508–509, Paris, France, October-November 1992.

[11] S. V. Pandit, "ECG baseline drift removal through STFT," in *Proceedings of 18th Annual International Conference of the Engineering in Medicine and Biology Society (EMBS '96)*, vol. 4, pp. 1405–1406, Amsterdam, The Netherlands, October-November 1996.

[12] R. F. von Borries, J. H. Pierluissi, and H. Nazeran, "Wavelet transform-based ECG baseline drift removal for body surface potential mapping," in *Proceedings of 27th Annual International Conference of the Engineering in Medicine and Biology Society (EMBS '05)*, pp. 3891–3894, Shanghai, China, September 2005.

[13] L. Xu, D. Zhang, and K. Wang, "Wavelet-based cascaded adaptive filter for removing baseline drift in pulse waveforms," *IEEE Transactions on Biomedical Engineering*, vol. 52, no. 11, pp. 1973–1975, 2005.

[14] S. Mallat, *A Wavelet Tour of Signal Processing*, Academic Press, San Diego, Calif, USA, 2nd edition, 1999.

[15] C. S. Burrus, R. A. Gopinath, and H. Guo, *Introduction to Wavelets and Wavelet Transforms, A Primer*, Prentice Hall, Englewood Cliffs, NJ, USA, 1998.

[16] I. Daubechies, *Ten Lecture on Wavelets*, SIAM, Philadelphia, Pa, USA, 1992.

[17] L. Schmorth, *An Introduction to Electrocardiography*, Oxford Press, Oxford, UK, 1986.

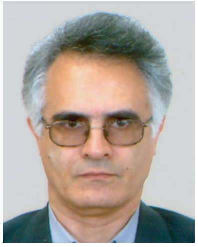

**Mohammad Ali Tinati** was born in 1953 in Iran. He received his B.S. degree (with high honor) in 1977, his M.S. degree in 1978 from Northeastern University, Boston, Mass, USA, and his Ph.D. degree from Adelaide University, Australia, in 1999. He had a long affiliation with the University of Tabriz, Iran. He served as an academic member of the Faculty of Electrical Engineering since 1979. His main research interests are biomedical signal processing and speech and image processing.

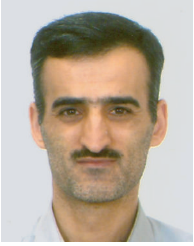

**Behzad Mozaffary** was born in 1968 in Iran. He received his B.S. degree in 1993 from University of Tabriz and his M.S. degree in 1996 from K.N.Toosi University of Technology. He is currently a Ph.D. candidate in electrical communication engineering in the University of Tabriz. His research interests are signal processing and speech processing.

